# Trajectories of medical service use among girls and boys with and without early-onset conduct problems

**DOI:** 10.3389/fpsyt.2022.915991

**Published:** 2023-01-04

**Authors:** Caroline E. Temcheff, Alexa Martin-Storey, Annie Lemieux, Eric Latimer, Michèle Déry

**Affiliations:** ^1^Department of Educational and Counselling Psychology, McGill University, Montreal, QC, Canada; ^2^Département de psychoéducation, Université de Sherbrooke, Sherbrooke, QC, Canada; ^3^Douglas Mental Health University Institute and McGill University, Montreal, QC, Canada

**Keywords:** conduct problems, conduct disorder, medical service use, injuries, psychiatric services, longitudinal, health service, youth

## Abstract

**Background:**

Children with conduct problems (CP) have been found to be heavy and costly medical service users in adulthood. However, there is little knowledge on how medical service use develops during childhood and adolescence among youth with and without childhood CP. Knowing whether differences in developmental trajectories of medical service use for specific types of problems (e.g., injuries) are predicted by childhood CP would help clinicians identify developmental periods during which they might intensify interventions for young people with CP in order to prevent later problems and associated increased service use.

**Methods:**

Participants were drawn from an ongoing longitudinal study of boys and girls with and without childhood CP as rated by parents and teachers. Medical service use was assessed using administrative data from a public single payer health plan. Latent growth modeling was used to estimate the mean trajectory of four types of medical visits (psychiatric, injury-related, preventative, total visits) across time and evaluate the effect of CP and other covariates.

**Results:**

Support the hypothesis that early CP predicts higher medical service use at nine years old, and that this difference persists in a chronic manner over time, even when controlling the effects of ADHD and family income. Girls had fewer medical visits for psychiatric reasons than boys at baseline, but this difference diminished over time.

**Conclusions:**

Clinicians should be aware that childhood CP already predicts increased medical service use in elementary school. Issues specific to different contexts in which injuries might occur and sex differences are discussed.

## Trajectories of medical service use among girls and boys with and without early-onset conduct problems

Conduct problems (CP) refer to symptoms of conduct disorder and/or oppositional defiant disorder, which are two frequently co-occurring mental health disorders (1, 2). These disorders are manifested in behaviors that violate the rights of others, and/or behaviors that bring the individual into conflict with societal norms (e.g., rule-breaking, aggression, lying, opposition to authority). In terms of developmental course, conduct disorder is considered to have an “early onset” if symptoms appear before age 10 (3), whereas oppositional defiant disorder usually appears before 8 years old (3).

Focusing on the developmental course of CP is important, as significant CP in childhood tend to persist into adolescence and adulthood and are linked with negative long-term consequences as well as physical and mental health problems (4–7). Childhood CP have been linked with internalizing disorders (8–11), addictive behaviors and disorders (9, 12–15), antisocial personality disorder (16, 17), high body mass index and obesity (14, 18), injuries (19–21), and sexually transmitted diseases (15). The fact that children with CP have been found to be heavy and costly medical service users in adulthood (22, 23) may be reflective of this increased prevalence of mental and physical health problems among people with childhood histories of CP.

The association between childhood CP and medical service utilization in adulthood has been established with longitudinal studies from several countries (14, 22–25). For example, individuals with childhood histories of CP were more likely to show patterns of increased medical service usage in adulthood, including medical visits due to injuries and lifestyle-related illnesses (e.g., alcoholism, obesity, or diabetes), mental health and counseling visits, visits to specialists, and visits to emergency departments (22, 23, 25, 26). This body of work suggests that childhood CP can have important consequences for later medical service use. However, there is little knowledge on how medical service use develops during childhood and adolescence among youth with and without childhood CP. The importance of examining the individual during transitional periods, such as adolescence, is particularly emphasized in the Life Course Health Development Framework (27) since this period of both vulnerability and plasticity is when individual trajectories are the most malleable to interventions (27). Knowing whether differences in trajectories of medical service use for different problems (e.g., injuries, psychiatric care) are predicted by childhood CP would help physicians and mental health professionals identify developmental periods during which they might increase their vigilance for certain problems or intensify interventions aimed at youth with CP. In so doing, they may be able to prevent problems and associated increased service use.

The extant literature suggests that there are already differences in medical service use among youth with CP in adolescence. Although most studies are cross-sectional, they show that children who met DSM criteria for CP usually have high physical and/or mental health service usage (28–32). Among the few longitudinal studies examining the association between childhood CP and later medical service use, youth with CP were more likely to use parent-reported outpatient counseling services, pediatric services and mental medical services (33) and to follow higher trajectories of outpatient and residential treatment trajectories (34). In a study using medical records to quantify past medical service use, parent-reported CP were prospectively linked with parent-reported service usage for injuries (such as lacerations, sprains, broken bones, head injuries, contusions, burns and poisonings) 1 year later, even after controlling for other family-level characteristics (35).

All these studies, with one exception (35), employ parental reports of service usage. However, even Bradbury and colleagues controlled for past service use using medical records, but the injury outcome variable was parent-reported. The accordance between parent and medical service use data varies across the type of medical service being assessed (36), and indicates that vulnerable families (e.g., families with high levels of poverty) report patterns of usage more divergent from medical records compared with other families (37), thus underscoring the importance of research employing medical records. Second, as alluded to earlier, most studies on this topic have been cross-sectional and do not take into account developmental context (e.g., including both children and adolescents in samples). However, it is possible that medical service needs change over time in youth. One study that considered trajectories of medical service use across childhood and adolescence based service use data on parental reports (34). Further research examining medical service use based on medical records over time is needed. Third, most studies generally provide a limited picture of service use, typically focusing on psychiatric and/or general pediatric service usage (30, 38). In order to better orient medical services for youth with CP, the types of problems for which services are needed should also be examined from a developmental perspective. In addition, it would be important to examine routine medical care (i.e., preventative visits). Indeed, early preventative care has been shown to be predictive of less CP in childhood (39–42), but how the presence of CP may shape trajectories of later preventative care in adolescence remains unclear.

The present study extends current knowledge by using longitudinal data on medical service usage from childhood through to adolescence (ages 9–16) and utilizing medical records to obtain a portrait of medical service use unbiased by retrospective self-reporting. This study evaluates the effects of childhood CP on trajectories of medical visits due to psychiatric reasons, visits for injuries, preventative visits, and total visits. In doing so, we also consider the impacts of attention symptoms (ADHD), socio-economic status, and child sex. Indeed, one of the most influential models of the development of CP, conceptualized by Moffitt (43, 44), posits that vulnerabilities at the individual level (e.g., ADHD) and vulnerabilities at the micro level (e.g., low socioeconomic status) increase the likelihood of developing and maintaining CP. These same factors are also associated with negative health outcomes (45–47). This would suggest controlling for these factors when exploring how CP shape medical service usage patterns. Particularly, it is important to disentangle the effects of ADHD and CP in the prediction of trajectories of medical visits for injuries, since ADHD has been related to injuries (48, 49) and injuries have been found to be a common reason why individuals with CP use medical services (22, 23, 26).

Though childhood CP are less frequent among girls than boys, CP that appear in elementary-aged girls seem at greater risk for persistence, at least until adolescence (50). Further, girls with CP are at greater risk for having comorbid mental health disorders (1, 51–53) and of exhibiting a variety of health-risk behaviors, such as risky sexual activities (7). Despite the fact that girls may have more persistent CP and more comorbidity, some studies have found that girls with CP are less likely than boys with CP to be referred for mental health services (54, 55). However, studies examining medical service use in adulthood have found that though adult women with a history of CP generally use more medical services than men, no significant interactions between sex and CP were found (22, 23, 26). This extant literature suggests a lower level of medical service use in childhood, but a steeper increase, at least for mental health reasons, among girls with CP. The current study therefore is well-poised to examine how sex may interact with CP to shape the use of medical services across childhood and adolescence among boys and girls with and without early CP.

The overarching goal of this study is to examine the predictive ability of early CP on trajectories of medical service use from childhood through to adolescence. Another objective is to verify if these predictive associations between early CP and later medical service use is the same for boys and girls. Based on theoretical models (27, 43, 44) and the extant literature, we hypothesize that childhood CP will be positively related to increased visits for psychiatric reasons, injuries, and overall medical service use, even when controlling for ADHD and low socio-economic status. Conversely, considering that children with CP live in families with greater levels of vulnerability (43, 44), we hypothesize that children with CP will receive fewer preventative medical services. Finally, we expected a steeper increase of medical service use from childhood to adolescence among girls.

## Methods

### Participants

Participants (*N* = 744; 47% girls) were drawn from an ongoing longitudinal study of boys and girls with and without childhood CP. Children were recruited between 2008 and 2010 across 155 public schools from four regions in Québec, Canada. They ranged from six to nine years old (*M* = 8.39, *SD* =.93) at the time of recruitment. Our selection methodology was aimed at increasing the probability of recruiting children with clinical levels of CP before age 10. Clinical levels of CP were operationalized as reaching the clinical cut-off (*T*-score ≥ 70) on either the DSM-oriented scale for Conduct Problems or Oppositional Defiant Problems of the *Achenbach System of Empirically Based Assessment* (ASEBA) (56, 57) by parents or teachers.

First, approximately half of the sample (*n* = 370) was selected from school board lists of children younger than 10 years who were referred to psychosocial school-based services for CP. To obtain approximately equal numbers of boys and girls, all girls and about one out of four boys were invited to participate. Of these, 274 children (44% girls) were classified as having clinical CP before the age of 10 years at the time of recruitment, and 29 others who had borderline levels of CP (i.e., T scores between 65 and 69) developed clinical levels of CP over the subsequent years (still prior to age 10). The remaining 67 children did not develop CP before age 10.

To address potential concerns regarding the recruitment of children with CP exclusively from those receiving services, and in order to recruit an appropriate comparison group (without CP), an additional 881 children who were not receiving services were screened for CP with same DSM-oriented scales by parents and teachers (56, 57). This multi-gated screening procedure was carried out in schools with a high deprivation index in order to reduce socio-economic disparities between children with and without clinical levels of CP and to increase the probability of identifying children with clinical levels of CP. Among these, 54 children (65% girls) were identified as having clinical levels of CP before age 10 at the time of recruitment. Forty-one children had borderline levels of CP at study inception and among these, 19 developed clinical levels of CP over the subsequent years, but still before age 10. The remaining twenty-two of these children did not develop clinical levels of CP. We also selected 279 children from this same pool who were not at-risk (T > 65) for CP at study inception according to parent and teacher ratings. Please see supplemental material for a graphic presentation of the sample selection.

As such, the total sample of children with early clinical CP is 376 children (46 % girls). Our final comparison group was comprised of all those children who never scored in the clinical level for CP by parents or teachers before 10 years old (*n* = 368).

Seven years later, in the eighth annual wave of assessment 685 participants (92%) gave their consent for the research team to gain access to their provincial medical records. Of the 685 participants, 348 had CP at or before age 10, and 337 children did not.

No statistical differences were observed between the 59 participants without medical service use data and the 685 participants included in this study in terms of age (*p* = 0.25), sex (*p* = 0.87), income group (*p* = 0.205), CP (*p* = 0.62) or ADHD (*p* = 0.62).

### Measures

#### Medical service utilization

The *Régie de l'assurance maladie du Québec* (RAMQ) holds administrative data on the persons registered and eligible for the Québec Health Insurance Plan, which is the single-payer public health plan. Data from the RAMQ will provide information on physician services billed on a fee-for-service basis (e.g., type of service, diagnosis).

In order to evaluate the services received by the participants, the number of annual visits between their 9th and 16th birthdays were used. Following our hypotheses, three types of medical visits were distinguished: psychiatric problems, injuries, and well-child visits. Visits for psychiatric reasons included all codes referring to psychiatrist visits, as well as those that refer to visits to other types of physicians that led to a psychiatric diagnosis (e.g., depression or anxiety) or treatment for psychiatric conditions (e.g., medication). Visits for injuries included codes referring to medical visits with diagnoses for injuries (i.e., bone breaks, burns, concussions, or cuts) or codes suggesting treatments for injuries (e.g., putting on a cast). Well-child visits were determined using the specific code meant for this type of routine medical check-up. In addition, we also examined the total number of yearly medical visits which included the three types of visits previously mentioned as well as visits for other reasons (e.g., infections and chronic conditions).

In total, 31 children moved away at some point between ages 9 and their 16th birthday (4.5%). These children's data were used only for the years during which they resided in Quebec. Given that not all participants had reached their 14th, 15th, or16th birthdays by the time data was extracted, only those participants who had completed three quarters of their year or older had their medical service utilization data prorated and included for the last quarter of the year. The data for the other participants were counted as missing for their last year of data. This prorating of the data was only carried out on the total number of medical visits per year, not on visits for specific reasons (i.e., injuries, psychiatric or well-child), because of low base rates of visits for specific reasons.

Given the above, the number of participants is slightly different for each age. In total, 685 participants had data at 9 years old, while 547 participants had data for the year between ages 15 and 16 (80%) (Table 1). Special attention was paid to the distribution of the data. Extreme values were truncated at the 98th percentile of the distributions across the 7 years on each of the medical service use measures.

**Table 1:** Means and standard deviations for medical visits by year and sex (*N* = 685).

				**Total**	**Boys No**	**Boys CP**	**Girls No**	**Girls CP**
				***n* = 685**	**CP *n* = 177**	***n* = 187**	**CP *n* = 160**	***n* = 161**
	**Valid N**	**Min**	**Max**	**Mean (SD)**	**Mean (SD)**	**Mean (SD)**	**Mean (SD)**	
Psy Act visits−9y	684	0	8	0.43 (1.56)	0.24 (1.31)	0.84 (2.05)	0.00 (0.00)	0.57 (1.80)
Psy Act visits−10y	682	0	8	0.37 (1.39)	0.18 (0.96)	0.80 (1.97)	0.00 (0.00)	0.46 (1.52)
Psy Act visits−11y	682	0	8	0.36 (1.39)	0.15 (0.82)	0.70 (1.91)	0.03 (0.25)	0.53 (1.71)
Psy Act visits−12y	681	0	8	0.40 (1.52)	0.09 (0.61)	0.79 (2.09)	0.03 (0.25)	0.65 (1.95)
Psy Act visits−13y	675	0	8	0.40 (1.45)	0.11 (0.72)	0.76 (1.94)	0.04 (0.26)	0.65 (1.88)
Psy Act visits−14y	642	0	8	0.38 (1.43)	0.12 (0.79)	0.50 (1.56)	0.14 (0.70)	0.78 (2.12)
Psy Act visits−15y	547	0	8	0.36 (1.36)	0.11 (0.77)	0.44 (1.45)	0.15 (0.82)	0.76 (2.02)
Total injury visits−9y	684	0	5	0.35 (1.01)	0.21 (0.71)	0.52 (1.22)	0.24 (0.90)	0.42 (1.09)
Total injury visits−10y	682	0	5	0.32 (0.98)	0.30 (0.91)	0.33 (1.03)	0.35 (0.98)	0.33 (1.01)
Total injury visits−11y	682	0	5	0.42 (1.12)	0.33 (0.98)	0.53 (1.32)	0.39 (1.16)	0.41 (0.96)
Total injury visits−12y	681	0	5	0.33 (0.98)	0.34 (1.06)	0.45 (1.16)	0.23 (0.79)	0.27 (0.80)
Total injury visits−13y	675	0	5	0.47 (1.20)	0.49 (1.27)	0.41 (1.09)	0.46 (1.16)	0.54 (1.26)
Total injury visits−14y	642	0	5	0.42 (1.12)	0.40 (1.10)	0.51 (1.29)	0.38 (1.02)	0.40 (1.01)
Total injury visits−15y	547	0	5	0.37 (1.05)	0.38 (1.11)	0.43 (1.15)	0.27 (0.96)	0.39 (0.95)
Regular visits−9y	684	0	3	0.19 (0.51)	0.23 (0.53)	0.20 (0.57)	0.19 (0.50)	0.12 (0.41)
Regular visits−10y	682	0	4	0.18 (0.55)	0.24 (0.59)	0.16 (0.60)	0.16 (0.44)	0.19 (0.54)
Regular visits−11y	682	0	4	0.19 (0.58)	0.22 (0.59)	0.16 (0.60)	0.19 (0.45)	0.19 (0.67)
Regular visits−12y	681	0	4	0.21 (0.63)	0.25 (0.61)	0.25 (0.81)	0.16 (0.47)	0.16 (0.53)
Regular visits−13y	675	0	4	0.22 (0.68)	0.16 (0.55)	0.29 (0.85)	0.22 (0.64)	0.18 (0.60)
Regular visits−14y	642	0	4	0.18 (0.60)	0.15 (0.53)	0.20 (0.69)	0.25 (0.70)	0.11 (0.45)
Regular visits−15y	547	0	4	0.18 (0.58)	0.14 (0.49)	0.22 (0.67)	0.20 (0.59)	0.14 (0.58)
Total visits−9y	684	0	25	4.03 (5.03)	3.22 (4.42)	5.34 (5.46)	3.06 (4.16)	4.35 (5.57)
Total visits−10y	682	0	25	3.85 (4.78)	3.09 (4.32)	5.04 (5.27)	3.19 (4.61)	3.95 (4.58)
Total visits−11y	682	0	25	4.05 (4.91)	3.17 (4.18)	5.34 (5.16)	3.40 (4.73)	4.18 (5.25)
Total visits−12y	681	0	25	4.00 (5.17)	3.17 (4.43)	5.31 (5.71)	3.27 (4.34)	4.12 (5.71)
Total visits−13y	675	0	25	4.12 (5.05)	3.06 (4.04)	5.02 (5.21)	3.68 (4.87)	4.71 (5.78)
Total visits−14y	642	0	25	4.26 (5.45)	3.03 (4.56)	4.59 (5.39)	4.48 (5.34)	5.07 (6.30)
Total visits−15y	547	0	25	4.28 (5.60)	3.17 (4.69)	4.16 (5.02)	4.23 (5.07)	5.75 (7.23)

#### Early CP

The presence of early CP was operationalized as scoring in the clinical range at or before age nine on either the DSM-oriented scale for Conduct Problems and Oppositional Defiant Problems from the parent-report *Child Behavioral Checklist* (CBCL) and *Teacher Report Form* (TRF) of the ASEBA (56, 57). Both scales contain five items assessing oppositional defiant behaviors, the CBCL contains 17 items that assess conduct behaviors and the TRF contains 13 items. All items are arranged on a 3-point Likert scale, ranging from 0 (*not true*) to 2 (*very true* or *very often*). Following age and sex appropriate norms of the instrument, raw scores were converted to *T*-scores. Participants were deemed to have CP if their scores were equal to or above the at-risk clinical range (*T* ≥ 70). A dichotomous score indicating presence or absence of CP was used in the subsequent analyses.

#### Covariates

Child sex was reported by parents at study inception. Family income and child ADHD symptomatology as reported by parents when the child was nine years old was also included as control variables. Family income was assessed based on primary caregiver report using a 14-point scale that was subsequently weighted to normalize the distribution. Median yearly household income for this sample was between 50,000$CAN and 59,000$CAN. Child ADHD symptomatology was also collected before age 10 from parents using the Conners ADHD/DSM-IV Scales—Parent version (CADS-P) (18 items: parent Cronbach's alpha= 0.94) (58). The t score related to the total score was used as a control variable.

### Procedure

Following approval for this study from the university's ethics review board, parental consent was obtained yearly prior to the collection of CP data. Data collection took place in the homes of the participants by trained graduate-level research assistants. In addition, parental consent was sought yearly so that information regarding child CP could be collected from the child's teacher. Families and teachers were financially compensated for their time. Seven years after study inception, participants, and their parents were asked for permission for the research team to obtain data on their medical service utilization. Approval to obtain provincial medical records of participants was then asked of the *Commission d'accès à l'information du Québec*, which is the regulatory body in Québec which oversees research access to public health records. Once this formal authorization was granted, the research team requested the data from the RAMQ.

### Statistical analysis

Latent growth modeling (LGM) was used to estimate the mean trajectory of the four types of services across time and evaluate the effect of CP and other covariates. LGM estimates the associations between longitudinal variables using the covariance matrix. This technique simultaneously calculates inter-person and intra-person variability. Each individual has their own trajectory and variability over time, just as each group has an average trajectory and average variability. In addition to inter-individual differences, this variability at the individual level also affects the shape (intercept and slope) of the mean trajectory.

The seven annual measurements (between 9th birthday and day before 16th birthday) of medical visits were used in the model as the longitudinal variables. In order to better suit the asymmetry of the distributions, square roots of all measures were calculated and a robust technique of maximum likelihood (MLR) for non-normal continuous data were used to estimate all models. All analyzes were performed with Mplus 7.4 software (59). For missing data, the option of estimating models via full information maximum likelihood was used. Across all 7 years, 95.8% of data were available for our participants.

The analyses were carried out in two steps. First, the base model was estimated in order to describe the average longitudinal trajectory of medical visits. For each of the medical service use type (i.e., overall medical service use, as well as use due to injuries, psychiatric problems, and well-child check-ups), the linear and quadratic trajectories were tested in order to identify which shape best represented the data. Then, the model was tested with CP, as well as the covariates and the interaction between child sex and CP.

In order to validate the models, the Chi-square statistic (X^2^), Root Mean Square Error of Approximation (RMSEA) (60) and Comparative Fit Index (CFI) (61) were used. In addition, the BIC statistic was used to identify the best model between the linear and quadratic trajectories. Indeed, the model with the smallest BIC was retained.

## Results

Descriptive and correlational analyses of the four types of services from ages 9–15 are presented in Tables 1, 2. All years of the different types of medical service use were correlated with other years of the same service in order to evaluate relative stability of scores (Table 2). As indicated by high correlations between scores at different measurement points, individuals generally retained their relative position across the years (62). The stability at the aggregate (group) level does not imply, however, that there is no change at the individual level. Individual rates of stability (or change) were examined next by growth curve analysis.

**Table 2:** Correlations (truncated scores).

	**9y**	**10y**	**11y**	**12y**	**13y**	**14y**	**15y**
Psy Act Visits							
9y	-	0.58***	0.56***	0.46***	0.44***	0.34***	0.20***
10y	0.58***	-	0.66***	0.51***	0.55***	0.40***	0.16***
11y	0.56***	0.66***	-	0.69***	0.60***	0.46***	0.21***
12y	0.46***	0.51***	0.69***	-	0.72***	0.62***	0.35***
13y	0.44***	0.55***	0.60***	0.72***	-	0.66***	0.39***
14y	0.34***	0.40***	0.46***	0.62***	0.66***	-	0.46***
15y	0.20***	0.16***	0.21***	0.35***	0.39***	0.46***	-
total injury visits	**9y**	**10y**	**11y**	**12y**	**13y**	**14y**	**15y**
9y	-	0.13**	0.10*	0.04	0.08*	0.06	0.09*
10y	0.13**	-	0.14***	0.10**	0.10*	0.11**	−0.02
11y	0.10*	0.14***	-	0.19***	0.08*	0.06	0.04
12y	0.04	0.10**	0.19***	-	0.06	0.10*	0.03
13y	0.08*	0.10*	0.08*	0.06	-	0.14***	0.08
14y	0.06	0.11**	0.06	0.10*	0.14***	-	0.18***
15y	0.09*	−0.02	0.04	0.03	0.08	0.18***	-
regular visits	**9y**	**10y**	**11y**	**12y**	**13y**	**14y**	**15y**
9y	-	0.38***	0.35***	0.27***	0.25***	0.31***	0.10*
10y	0.38***	-	0.37***	0.35***	0.26***	0.29***	0.17***
11y	0.35***	0.37***	-	0.29***	0.29***	0.27***	0.09*
12y	0.27***	0.35***	0.29***	-	0.40***	0.23***	0.24***
13y	0.25***	0.26***	0.29***	0.40***	-	0.38***	0.30***
14y	0.31***	0.29***	0.27***	0.23***	0.38***	-	0.17***
15y	0.10*	0.17***	0.09*	0.24***	0.30***	0.17***	-
total visits	**9y**	**10y**	**11y**	**12y**	**13y**	**14y**	**15y**
9y	-	0.52***	0.40***	0.39***	0.31***	0.26***	0.31***
10y	0.52***	-	0.58***	0.42***	0.40***	0.35***	0.32***
11y	0.40***	0.58***	-	0.49***	0.43***	0.33***	0.29***
12y	0.39***	0.42***	0.49***	-	0.51***	0.44***	0.36***
13y	0.31***	0.40***	0.43***	0.51***	-	0.54***	0.41***
14y	0.26***	0.35***	0.33***	0.44***	0.54***	-	0.47***
15y	0.31***	0.32***	0.29***	0.36***	0.41***	0.47***	-

*p < 0.05; **p < 0.01; ***p < 0.001.

### Latent growth modeling

All unconditional models suggested a linear trajectory as evidenced by the lowest BIC model and non-significant quadratic growth estimators (mean and variance). The average slope was non-significant for all of the base trajectories, meaning that on average the number of visits was stable across time. All four models presented significant variance in both intercept and slope parameters which supports the addition of predictors to the model in order to better explain the trajectories. The results of the four base models are presented in Table 3.

**Table 3:** LGM base models (*n* = 685).

	**Psy act visits**			**Total injury visits**		
	**Chi-Square** = **42.079**, ***df*** = **21**, ***p*** = **0.004 RMSEA** = **0.038**			**Chi-Square** = **19.118**, ***df*** = **25**, ***p*** = **0.792 RMSEA** = **0.000**		
	**b**	**Se**	***p-value***	**b**	**Se**	***p-value***
Intercept (I)	1.111	0.015	0.000	1.115	0.009	0.000
Slope (S)	0.000	0.003	0.872	0.004	0.003	0.161
Variance (I)	0.115	0.020	0.000	0.016	0.005	0.002
Variance (S)	0.004	0.001	0.000	0.001	0.000	0.024
	**Regular visits chi-square** = **30.091**, ***df*** = **21**, ***p*** = **0.090 RMSEA** = **0.025**			**Total visits chi-square** = **42.276**, ***df*** = **22**, ***p*** = **0.006 RMSEA** = **0.037**		
	**b**	**Se**	***p-value***	**b**	**Se**	***p-value***
Intercept (I)	1.074	0.006	0.000	2.010	0.032	0.000
Slope (S)	-0.001	0.001	0.388	0.008	0.007	0.251
Variance (I)	0.016	0.004	0.000	0.484	0.048	0.000
Variance (S)	0.000	0.000	0.034	0.015	0.002	0.000

Results for the conditional models with child sex, the presence of CP at or before age 9, and their interaction are shown in Table 4. Control variables included family income and ADHD symptoms. All models showed good fit.

**Table 4:** LGM with predictors.

	**Psy act visits**			**Total injury visits**		
	**Chi-Square** = **84.954**, ***df*** = **46**, ***p*** = **0.000 RMSEA** = **0.035**			**Chi-Square** = **41.958**, ***df*** = **50**, ***p*** = **0.784 RMSEA** = **0.000**		
	**CFI** = **0.035**			**CFI** = **1.000**		
	**b**	**Se**	***p*-value**	**b**	**Se**	***p*-value**
Intercept	0.807	0.086	0.000	1.175	0.059	0.000
Income	0.002	0.004	0.678	-0.005	0.003	0.057
ADHD	0.004	0.001	0.001	-0.001	0.001	0.282
Sex	-0.077	0.021	0.000	0.012	0.022	0.606
CP	0.135	0.045	0.003	0.064	0.029	0.028
Sex x CP	-0.071	0.054	0.189	-0.030	0.037	0.425
Slope	-0.009	0.018	0.611	-0.019	0.016	0.246
Income	-0.001	0.001	0.439	0.000	0.001	0.526
ADHD	0.000	0.000	0.602	0.000	0.000	0.075
Sex	0.013	0.005	0.007	-0.007	0.006	0.248
CP	-0.012	0.009	0.201	-0.014	0.008	0.096
Sex x CP	0.016	0.012	0.181	0.006	0.010	0.573
	**Regular visits chi-square** = **64.622**, ***df*** = **46**, ***p*** = **0.036 RMSEA** = **0.024**			**Total visits chi-square** = **61.335**, ***df*** = **47**, ***p*** = **0.078 RMSEA** = **0.021**		
	**CFI** = **0.958**			**CFI** = **0.990**		
	**b**	**Se**	***p*** **-value**	**b**	**Se**	***p*** **-value**
Intercept	1.077	0.037	0.000	1.355	0.195	0.000
Income	0.003	0.002	0.089	-0.001	0.009	0.888
ADHD	0.000	0.001	0.909	0.009	0.003	0.002
Sex	-0.024	0.017	0.154	-0.094	0.084	0.261
CP	-0.021	0.020	0.285	0.359	0.094	0.000
Sex x CP	0.020	0.026	0.434	-0.293	0.124	0.018
Slope	0.001	0.008	0.902	-0.089	0.042	0.033
Income	-0.001	0.000	0.216	0.001	0.002	0.765
ADHD	0.000	0.000	0.584	0.001	0.001	0.042
Sex	0.008	0.004	0.054	0.054	0.018	0.003
CP	0.008	0.004	0.063	-0.034	0.021	0.107
Sex x CP	-0.011	0.006	0.049	-0.001	0.028	0.964

#### Medical visits for psychiatric reasons

The longitudinal trajectory of medical service utilization for psychiatric reasons shows that the presence of CP was associated with more visits for psychiatric reasons at age nine (*b* = 0.135, *p* = 0.003) and this difference is maintained up to age 15 given the non-significance of the effect of CP on the slope. The presence of ADHD was also associated with more visits at age nine (*b* = 0.004, *p* = 0.001), which was also maintained over time. Girls, on average, had fewer medical visits for psychiatric reasons than boys at baseline (*b* = −0.077, *p* = 0.000), but this difference diminishes over time (*b* = 0.013, *p* = 0.007).

#### Visits for injuries

Childhood CP was the only variable associated with more medical visits for injuries at age nine (*b* = 0.064, *p* = 0.028), and this higher level of injuries among children with CP persisted over time.

#### Preventative visits

The latent growth trajectory of annual well-child visits did not show any difference between boys and girls at baseline (9 years old) nor between children with and without CP. However, results showed that boys without CP had fewer preventative visits across time than boys with CP according the significant interaction on the slope of the trajectory (*b* = −0.011, *p* = 0.049), while no differences were found between girls with and without CP across time.

#### Total medical visits

In terms of the total number of medical services received at baseline, boys, and girls with CP received more services than those without (*b* = 0.359, *p* = 0.000). In addition, at 9 years old, a moderation effect between sex and CP was also found (*b* = −0.293, *p* = 0.018), suggesting that boys with CP had even higher initial levels of service use than girls with CP. However, for children without CP, no differences were observed between boys and girls in terms of their initial levels of service use. An effect for ADHD was also found at baseline (*b* = 0.009, *p* = 0.002), suggesting that the presence of ADHD was positively associated with more services.

**Figure 1:**
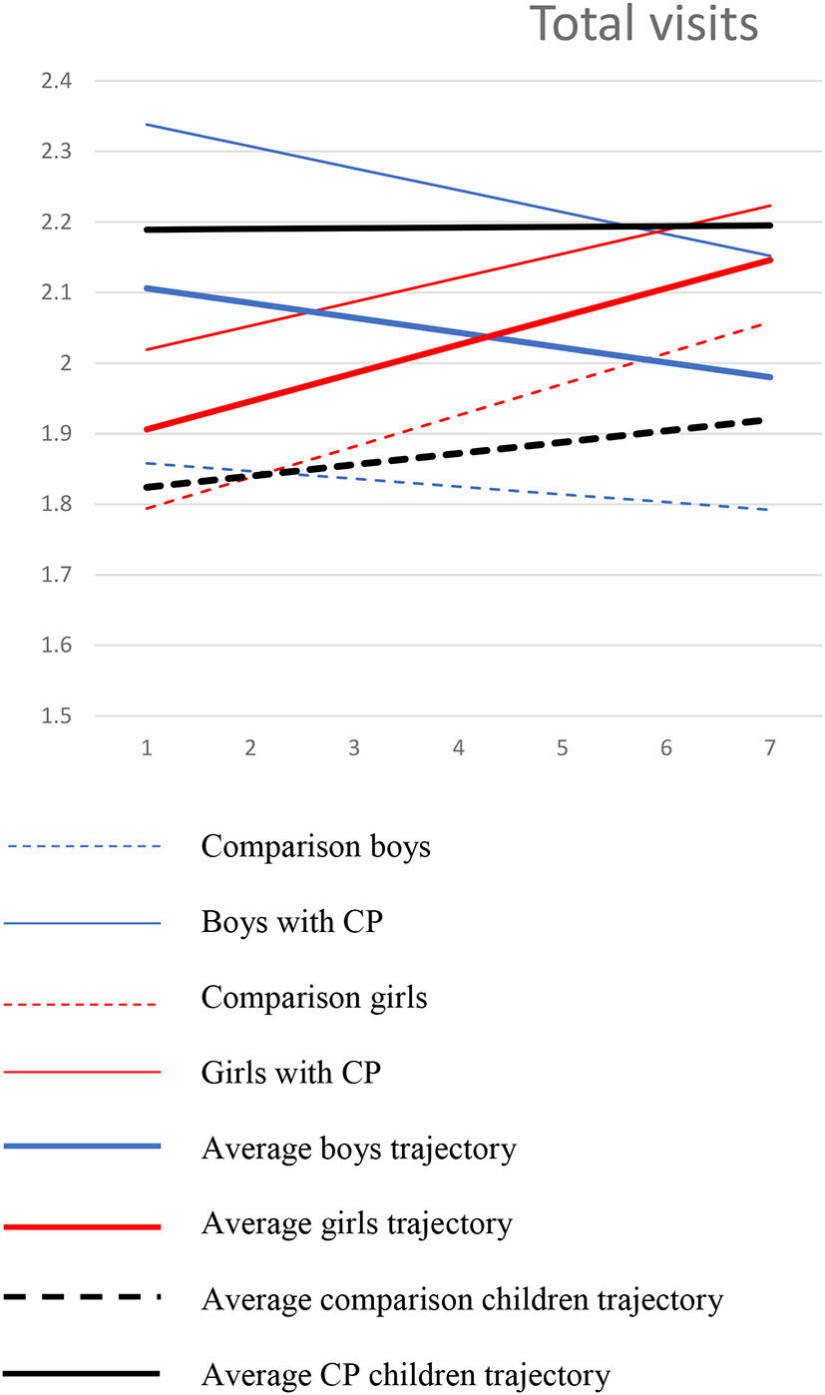
Trajectories of total visits among boys and girls with and without CP.

The results on the slope of the trajectory show that in addition to presenting more services at age nine, the presence of ADHD symptomatology increases medical service utilization over time (*b* = 0.001, *p* = 0.042). Girls also increased their medical service use over time (*b* = 0.054, *p* = 0.003). See Figure 1.

## Discussion

This study aimed to examine how CP influences the evolution of medical service use so that physicians and mental health practitioners may better anticipate the needs of children with CP. In examining medical records between children's ninth and the day before their sixteenth birthdays, we observed three main findings. First, in line with our hypotheses, we observed an effect of CP on the medical service utilization of children at nine years old, which was maintained over time until mid-adolescence. This effect was observed for visits for psychiatric reasons, for injuries, and for overall medical care. These findings show that the higher service use rates observed in adulthood (14, 22–25), are already present in mid-childhood and adolescence and suggests that childhood CP is a good predictor of chronically higher medical service use. These results were found even after controlling for family income and ADHD. Notably, in terms of disentangling the effects of CP and ADHD on injuries, CP appears to be a better predictor of injuries at nine years old and the chronicity of visits for injuries than ADHD. Given this novel finding, future research should investigate whether this may be due to lifestyle (e.g., family context or risk-taking) or relational (e.g., hostility or aggression) factors which may be more related to CP in childhood, rather than impulsivity, which may be common to both CP and ADHD.

Second, children with CP were not found to be different from children without CP in terms of their preventative care at nine years old. Longitudinally, the mean number of preventative visits among children in our sample was very low (average between 0.18 and 0.22 visits per year), while good practice suggests one preventative (or well-child) visit per year (63). This low use of preventative care is seen despite the fact that children with CP use more (non-preventative) services than those without, suggesting that they may benefit from increase preventative care. However, over time, boys with CP were found to slightly increase their use of preventative medical services compared to boys without CP. Taken together, these results suggest that particular attention should be paid to children with CP in terms of offering preventative care.

Third, as expected, a main effect for sex was found such that girls had fewer medical visits for psychiatric reasons than boys at nine years old, but this difference diminished over time until mid-adolescence. Whether this difference in service utilization is due to differences in service needs, or is related to lower referral rates for psychiatric services, which have been previously documented among young girls (particularly with CP) (54, 55), cannot be determined given the current design, but deserves future research attention.

In addition to the sex moderation effect found for preventative visits, a moderation effect of sex was also found for total medical visits. Specifically, at 9 years old, boys with CP had even higher levels of overall service utilization than girls with CP. However, among children without CP, no differences were observed between boys and girls. Given this result, mental health professionals and physicians working with young boys with clinical CP in middle childhood, should anticipate their increased medical service needs and should consider intensifying intervention at this developmental time in order to try to meet their needs.

The use of medical records to assess children's medical service use certainly presents advantages, including minimizing social desirability or recall biases. However, the use of medical administrative data may also present some shortcomings. For example, this type of data does not allow for the fine-grained analysis based on the context in which the injury occurred, such as a sport-related injury, or an injury related to aggressive or impulsive behavior. Complementary research utilizing parental reports could assist in clarifying the picture regarding the reasons for the increased injuries among children with CP. In addition, although our design was sufficiently powered to detect effects in terms of total numbers of injuries, we were unable to break this variable down further to investigate trajectories of types of injuries (e.g., head injuries, burns, broken bones etc). Replication with larger samples offering increased power could offer additional information as to the types of injuries these children may be experiencing. Finally, whether early intervention for childhood CP may alter trajectories of medical service use could not be examined given the current design, but would be an important direction for future research and could shed light on possible avenues to reducing medical service use and associated costs.

In summary, our results support the hypothesis that CP already predicts higher medical service use at 9 years old, and that this difference persists in a chronic manner over time. Since this result was found for psychiatric visits, visits for injuries, as well as for the total number of visits, clinicians should be particularly vigilant to different types of comorbid medical or mental health problems when treating children with early CP. Specifically for injuries, given that CP was predictive of increased medical service use over and above the effects of ADHD, clinicians should also consider different contexts in which injuries could occur among children with CP either because of the child's own characteristics, or because their environments may present specific risk factors for injuries (e.g., parental antisocial personality or neglect) (43, 44). In addition, our results echo other studies which have suggested that specific attention should be paid to girls (both with and without CP) in terms of mental health needs and associated service support. Of course, the recommendations made here rely on the availability of a highly trained behavioral health workforce and easy access to mental and behavioral health services for children with CP and their families. Finally, given the low rate of preventative care, and the fact that typically parents present their children for routine check-ups, physicians could be more proactive in ensuring that children with CP attend routine preventative care, particularly in early elementary school. More research investigating mediators of the increased medical service use among children with CP, as well as the potential moderating effects of life contexts are needed in order to better understand how to prevent child suffering and related increased medical service use.

## Data availability statement

Given the ethical agreements we have signed with the *Régie de l"assurance maladie du Quebec*, we are not permitted to share any of the medical service use data at any time, even when anonymized. However, we could share our syntaxes for the data analysis, upon reasonable request. Requests to access the syntaxes should be directed to CT at caroline.temcheff@mcgill.ca.

## Ethics statement

The studies involving human participants were reviewed and approved by Université de Sherbrooke Ethics Review Board. Written informed consent to participate in this study was provided by the participants' legal guardian/next of kin.

## Author contributions

CT: conceptualized the study, obtained the funding, obtained the data, oversaw data cleaning and analysis, and wrote the first draft. AM-S: contributed to study conceptualization and revised the drafts. AL: performed all analyses, revised methods, and results sections. EL: contributed to study conceptualization, assisted in data cleaning, and revised the drafts. MD: conceptualized the original longitudinal study, as well as the data collection used in this paper, contributed to writing the paper, and revising the drafts. All authors contributed to the article and approved the submitted version.

## References

[1] Costello EJ Mustillo S Erkanli A Keeler G Angold A. Prevalence and development of psychiatric disorders in childhood and adolescence. Arch Gen Psychiatr. (2003) 60:837–44. 10.1001/archpsyc.60.8.837 12912767

[2] Maughan B Rowe R Messer J Goodman R Meltzer H. Conduct disorder and oppositional defiant disorder in a national sample: developmental epidemiology. J Child Psychol Psychiatr. (2004) 45:609–21. 10.1111/j.1469-7610.2004.00250.x 15055379

[3] American Psychiatric Association. Diagnostic and Statistical Manual of Mental Disorders (DSM-5^®^).. In: *American Psychiatric Pub*. (2013). 10.1176/appi.books.97808904255968723190

[4] Kretschmer T Hickman M Doerner R Emond A Lewis G Macleod J. Outcomes of childhood conduct problem trajectories in early adulthood: findings from the ALSPAC study. Eur Child Adolescent Psychiatr. (2014) 23:539–49. 10.1007/s00787-013-0488-5 24197169 PMC4172989

[5] Lichtenstein P Cederlöf M Lundström S D'Onofrio BM Anckarsäter H Larsson H. Associations between conduct problems in childhood and adverse outcomes in emerging adulthood: a longitudinal Swedish nationwide twin cohort. J Child Psychol Psychiatr. (2020) 61:798–806. 10.1111/jcpp.13169 31849046 PMC7384167

[6] Moore AA Silberg JL Roberson-Nay R Mezuk B. Life course persistent and adolescence limited conduct disorder in a nationally representative US sample: prevalence, predictors, and outcomes. Soc Psychiatry Psychiatr Epidemiol. (2017) 52:435–43. 10.1007/s00127-017-1337-5 28180930 PMC5382064

[7] Odgers CL Moffitt TE Broadbent JM Dickson N Hancox RJ Harrington H. Female and male antisocial trajectories: from childhood origins to adult outcomes. Dev Psychopathol. (2008) 20:673–716. 10.1017/S0954579408000333 18423100

[8] Boutin S Roy V St-Pierre RA Déry M Lemelin JP Martin-Storey A. The longitudinal association between externalizing and internalizing problems: an exploration of the dual failure model. Dev Psychol. (2020) 56:1372–84. 10.1037/dev0000935 32352825

[9] Reef J Diamantopoulou S van Meurs I Verhulst FC van der Ende J. Developmental trajectories of child to adolescent externalizing behavior and adult DSM-IV disorder: results of a 24-year longitudinal study. Soc Psychiatr Psychiatr Epidemiol. (2011) 46:1233–41. 10.1007/s00127-010-0297-9 20936464 PMC3214259

[10] Déry M Lapalme M Jagiellowicz J Poirier M Temcheff C Toupin J. Predicting depression and anxiety from oppositional defiant disorder symptoms in elementary school-age girls and boys with conduct problems. Child Psychiatry Hum Dev. (2017) 48:53–62. 10.1007/s10578-016-0652-5 27209374

[11] Poirier M Temcheff CE Déry M Toupin J Verlaan P Lemelin JP. The role of academic skills in the evolution of conduct problems and depressive symptoms among children with and without early clinically significant conduct problems. J Early Adolesc. (2019) 39:340–70. 10.1177/0272431618757679

[12] Bardone AM Moffitt TE Caspi A Dickson N Silva PA. Adult mental health and social outcomes of adolescent girls with depression and conduct disorder. Dev Psychopathol. (1996) 8:811–29. 10.1017/S0954579400007446

[13] Temcheff CE Déry M St-Pierre RA Laventure M Lemelin J-P. Precocious initiation into smoking, alcohol use, and gambling among children with conduct problems. Can J Psychiatr Revue Can De Psychiatr. (2016) 61:50–8. 10.1177/0706743715620402 27582453 PMC4756600

[14] Wertz J Agnew-Blais J Caspi A Danese A Fisher HL Goldman-Mellor S. From childhood conduct problems to poor functioning at age 18 years: examining explanations in a longitudinal cohort study. J Am Acad Child Adolescent Psychiatr. (2018) 57:54–60.e4. 10.1016/j.jaac.2017.09.437 29301670 PMC5772703

[15] Bardone AM Moffitt TE Caspi A Dickson N Stanton WR Silva PA. Adult physical health outcomes of adolescent girls with conduct disorder, depression, and anxiety. J Am Acad Child Adolesc Psychiatry. (1998) 37:594–601. 10.1097/00004583-199806000-00009 9628079

[16] Moffitt TE Arseneault L Jaffee SR Kim-Cohen J Koenen KC Odgers CL. DSM-V conduct disorder: research needs for an evidence base. J CHild Psychol Psychiatr. (2007) 49:3–33. 10.1111/j.1469-7610.2007.01823.x 18181878 PMC2822647

[17] Robins LN. Sturdy childhood predictors of antisocial behavior: replications from longitudinal studies. Psychol Med. (1978) 8:611–22. 10.1017/S0033291700018821 724874

[18] Duarte CS Sourander A Nikolakaros G Pihlajamaki H Helenius H Piha J. Child mental health problems and obesity in early adulthood. J Pediatr. (2010) 156:93–7. 10.1016/j.jpeds.2009.06.066 19783001 PMC3586427

[19] Bijur PE Stewart-Brown S Butler N. Child behavior and accidental injury in 11,966 preschool children. Am J Dis Child. (1986) 140:487–92. 10.1001/archpedi.1986.02140190097036 3962946

[20] Bussing R Menvielle E Zima B. Relationship between behavioral problems and unintentional injuries in US children. Findings of the 1988 National Health Interview Survey. Arch Pediatr Adolescent Med. (1996) 150:50–6. 10.1001/archpedi.1996.02170260054009 8542007

[21] Rowe R Maughan B Goodman R. Childhood psychiatric disorder and unintentional injury: findings from a national cohort study. J Pediatr Psychol. (2004) 29:119–30. 10.1093/jpepsy/jsh015 15096533

[22] Temcheff CE Serbin LA Martin-Storey A Stack DM Hastings P Ledingham J. Childhood aggression, withdrawal and likeability, and the use of health care later: a longitudinal study. CMAJ. (2011) 183:2095–101. 10.1503/cmaj.091830 22083681 PMC3255142

[23] Temcheff CE Serbin LA Martin-Storey A Stack DM Ledingham J Schwartzman AE. Predicting adult physical health outcomes from childhood aggression, social withdrawal and likeability: a 30-year prospective, longitudinal study. Int J Behav Med. (2011) 18:5–12. 10.1007/s12529-010-9082-0 20383621

[24] D'Amico F Knapp M Beecham J Sandberg S Taylor E Sayal K. Use of services and associated costs for young adults with childhood hyperactivity/conduct problems: 20-year follow-up. Br J Psychiatr. (2014) 204:441–7. 10.1192/bjp.bp.113.131367 24676966

[25] Herrenkohl TI Kosterman R Mason WA Hawkins JD McCarty CA McCauley E. Effects of childhood conduct problems and family adversity on health, health behaviors, and service use in early adulthood: tests of developmental pathways involving adolescent risk taking and depression. Dev Psychopathol. (2010) 22:655–65. 10.1017/S0954579410000349 20576185 PMC2892805

[26] Rivenbark JG Odgers CL Caspi A Harrington H Hogan S Houts RM. The high societal costs of childhood conduct problems: evidence from administrative records up to age 38 in a longitudinal birth cohort. J Child Psychol Psychiatry. (2018) 59:703–10. 10.1111/jcpp.12850 29197100 PMC5975095

[27] Halfon N Hochstein M. Life course health development: an integrated framework for developing health, policy, and research. The Milbank Quart. (2002) 80:433–79. 10.1111/1468-0009.00019 12233246 PMC2690118

[28] Offord DR Boyle MH Szatmari P Rae-Grant NI Links PS Cadman DT. Ontario child health study. II Six-month prevalence of disorder and rates of service utilization. Arch General Psychiatr. (1987) 44:832–6. 10.1001/archpsyc.1987.01800210084013 3498458

[29] Sawyer MG Miller-Lewis LR Clark JJ. The mental health of 13–17 year-olds in australia: findings from the national survey of mental health and well-being. J Youth Adolesc. (2007) 36:185–94. 10.1007/s10964-006-9122-x

[30] Wu P Hoven CW Bird HR Moore RE Cohen P Alegria M. Depressive and disruptive disorders and mental health service utilization in children and adolescents. J Am Acad Child Adolescent Psychiatr. (1999) 38:1081–90. 10.1097/00004583-199909000-00010 10504806

[31] Zahner GE Daskalakis C. Factors associated with mental health, general health, and school-based service use for child psychopathology. Am J Public Health. (1997) 87:1440–8. 10.2105/AJPH.87.9.1440 9314794 PMC1380967

[32] Georgiades K Duncan L Wang L Comeau J Boyle MH Ontario Child Health Study Team. Six-month prevalence of mental disorders and service contacts among children and youth in Ontario: evidence from the 2014 Ontario Child Health Study. Can J Psychiatry. (2019) 64:246–55. 10.1177/0706743719830024 30978138 PMC6463361

[33] Ford T Hamilton H Meltzer H Goodman R. Child mental health is everybody's business: the prevalence of contact with public sector services by type of disorder among british school children in a three-year period. Child Adolesc Ment Health. (2007) 12:13–20. 10.1111/j.1475-3588.2006.00414.x 32811033

[34] Okado Y Ewing E Rowley C Jones DE. Trajectories of mental health-related service use among adolescents with histories of early externalizing problems. J Adolescent Health. (2017) 61:198–204. 10.1016/j.jadohealth.2017.02.012 28438524 PMC5529137

[35] Bradbury K Janicke DM Riley AW Finney JW. Predictors of unintentional injuries to school-age children seen in pediatric primary care. J Pediatr Psychol. (1999) 24:423–33. 10.1093/jpepsy/24.5.423 10554454

[36] Murray KD El-Mohandes AAE El-Khorazaty MN Kiely M. Low-income minority mothers' reports of infant health care utilisation compared with medical records. Paediatr Perinat Epidemiol. (2007) 21:274–83. 10.1111/j.1365-3016.2007.00800.x 17439537

[37] Stone KE Burrell L Higman SM McFarlane E Fuddy L Sia C. Agreement of injury reporting between primary care medical record and maternal interview for children aged 0-3 years: implications for research and clinical care. Ambulat Pediatr. (2006) 6:91–5. 10.1016/j.ambp.2005.10.003 16530145

[38] Shivram R Bankart J Meltzer H Ford T Vostanis P Goodman R. Service utilization by children with conduct disorders: findings from the 2004 Great Britain child mental health survey. Eur Child Adolescent Psychiatr. (2009) 18:555–63. 10.1007/s00787-009-0012-0 19353233

[39] Eckenrode J Campa MI Luckey D Henderson CR Jr Cole R Kitzman H. Long-term effects of prenatal and infancy nurse home visitation on the life course of youths: 19-year follow-up of a randomized trial. Arch Pediatr Adolescent Med. (2010) 164:9–15. 10.1001/archpediatrics.2009.240 20048236

[40] Gardner F Connell A Trentacosta CJ Shaw DS Dishion TJ Wilson MN. Moderators of outcome in a brief family-centered intervention for preventing early problem behavior. J Consult Clin Psychol. (2009) 77:543–53. 10.1037/a0015622 19485594 PMC2793096

[41] Kitzman HJ Olds DL Cole RE Hanks CA Anson EA Arcoleo KJ. Enduring effects of prenatal and infancy home visiting by nurses on children: follow-up of a randomized trial among children at age 12 years. Arch Pediatr Adolescent Med. (2010) 164:412–8. 10.1001/archpediatrics.2010.76 20439791 PMC4225617

[42] Olds D Henderson CR Cole R Eckenrode J Kitzman H Luckey D. Long-term effects of nurse home visitation on children's criminal and antisocial behavior: 15-year follow-up of a randomized controlled trial. JAMA. (1998) 280:1238–44. 10.1001/jama.280.14.1238 9786373

[43] Moffitt TE. Adolescence-limited and life-course-persistent antisocial behavior: a developmental taxonomy. Psychol Rev. (1993) 100:674–701. 10.1037/0033-295X.100.4.674 8255953

[44] Moffitt TE. Life-course-persistent versus adolescence-limited antisocial behavior. In: Developmental Psychopathology. Hoboken, NJ: John Wiley & Sons, Inc. (2015). p. 570–98. 10.1002/9780470939406.ch15

[45] Costello EJ Compton SN Keeler G Angold A. Relationships between poverty and psychopathology: a natural experiment. JAMA. (2003) 290:2023–9. 10.1001/jama.290.15.2023 14559956

[46] Hanson MD Chen E. Socioeconomic status and health behaviors in adolescence: a review of the literature. J Behav Med. (2007) 30:263–85. 10.1007/s10865-007-9098-3 17514418

[47] Melchior M Moffitt TE Milne BJ Poulton R Caspi A. Why do children from socioeconomically disadvantaged families suffer from poor health when they reach adulthood? A life-course study. Am J Epidemiol. (2007) 166:966–74. 10.1093/aje/kwm155 17641151 PMC2491970

[48] Brehaut JC Miller A Raina P McGrail KM. Childhood behavior disorders and injuries among children and youth: a population-based study. Pediatrics. (2003) 111:262–9. 10.1542/peds.111.2.262 12563049

[49] Pastor PN Reuben CA. Identified attention-deficit/hyperactivity disorder and medically attended, nonfatal injuries: US school-age children, 1997-2002. Ambulatory Pediatr. (2006) 6:38–44. 10.1016/j.ambp.2005.07.002 16443182

[50] Brennan LM Shaw DS. Revisiting data related to the age of onset and developmental course of female conduct problems. Clin Child Fam Psychol Rev. (2013) 16:35–58. 10.1007/s10567-012-0125-8 23076722 PMC3684009

[51] Bernhard A Martinelli A Ackermann K Saure D Freitag CM. Association of trauma, posttraumatic stress disorder and conduct disorder: a systematic review and meta-analysis. Neurosci Biobehav Rev. (2018) 91:153–69. 10.1016/j.neubiorev.2016.12.019 28017839

[52] Morcillo C Duarte CS Sala R Wang S Lejuez CW Kerridge BT. Conduct disorder and adult psychiatric diagnoses: associations and gender differences in the US adult population. J Psychiatr Res. (2012) 46:323–30. 10.1016/j.jpsychires.2011.10.012 22172996 PMC3288967

[53] Stringaris A Lewis G Maughan B. Developmental pathways from childhood conduct problems to early adult depression: findings from the ALSPAC cohort. Br J Psychiatr. (2014) 205:17–23. 10.1192/bjp.bp.113.134221 24764545 PMC4076653

[54] Costello EJ He J Sampson NA Kessler RC Merikangas KR. Services for adolescents with psychiatric disorders: 12-month data from the National Comorbidity Survey-Adolescent. Psychiatr Services. (2014) 65:359–66. 10.1176/appi.ps.201100518 24233052 PMC4123755

[55] Verlaan P Déry M Temcheff CE Toupin J. Longitudinal determinants of school-based mental health service use for girls and boys with externalizing behavior problems. School Mental Health. (2018) 10:322–37. 10.1007/s12310-018-9249-4

[56] Achenbach TM. DSM-Oriented Guide for the Achenbach System of Empirically Based Assessment (ASEBA). (2013). p. 31–9.

[57] Achenbach TM Rescorla LA. Manual for the ASEBA Preschool Forms and Profiles. Burlington, VT: University of Vermont, Research center for children, youth (2000).

[58] Conners CK. Conners' Rating Scales - Revised: Technical Manual. North Tonawanda, NY: MHS (2001).

[59] Muthén L Muthén B. Mplus user's Guide, 7th Edn. Los Angeles. CA: Muthén and Muthén (1998).

[60] Browne MW Cudeck R. Alternative ways of assessing model fit. Sociol Methods Res. (1992) 21:230–58. 10.1177/0049124192021002005

[61] Bentler PM. Comparative fit indexes in structural models. Psychol Bull. (1990) 107:238–46. 10.1037/0033-2909.107.2.238 2320703

[62] Deković M Buist KL Reitz E. Stability and changes in problem behavior during adolescence: latent growth analysis. J Youth Adolescence. (2004) 33:1–12. 10.1023/A:1027305312204

[63] Hagan JF Shaw JS Duncan PM. Bright futures: guidelines for health supervision of infants, children, and adolescents. Am Acad Pediatr. (2007). 10.1542/97815811022399430154

