# Supplementary material for: Trajectories of medical service use among girls and boys with and without early-onset conduct problems
Source: Front Psychiatry. 2023 Jan 4;13:915991. doi: 10.3389/fpsyt.2022.915991 (PMC9846218; doi:10.3389/fpsyt.2022.915991)
Supplement: Supplementary file 1 [file Presentation_1.pptx]

## Slide 1
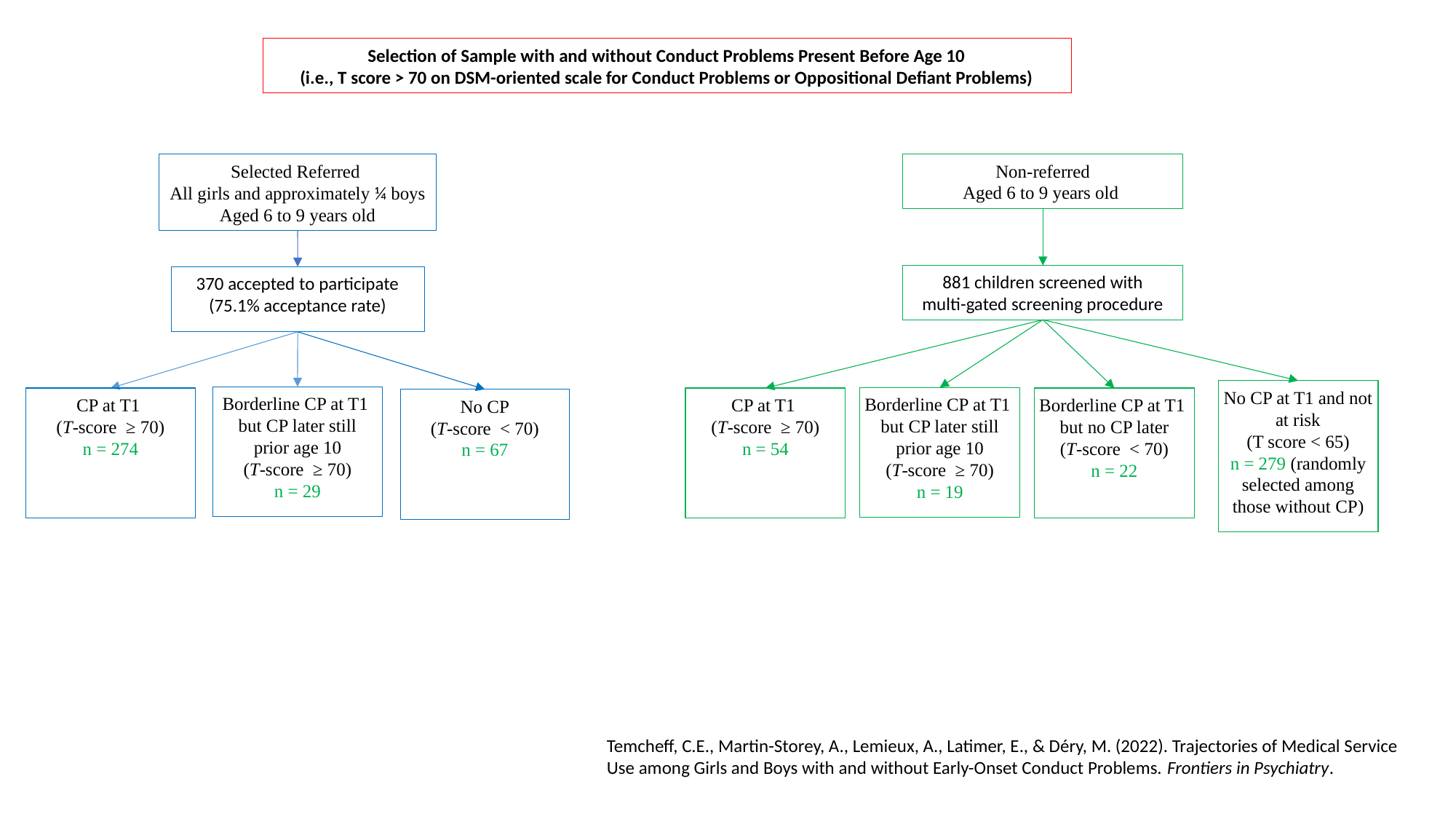

Selection of Sample with and without Conduct Problems Present Before Age 10
(i.e., T score > 70 on DSM-oriented scale for Conduct Problems or Oppositional Defiant Problems)
Non-referred
Aged 6 to 9 years old
Selected Referred
All girls and approximately ¼ boys
Aged 6 to 9 years old
881 children screened with
multi-gated screening procedure
370 accepted to participate
(75.1% acceptance rate)
No CP at T1 and not at risk
(T score < 65)
n = 279 (randomly selected among those without CP)
Borderline CP at T1
but CP later still
prior age 10
(T-score ≥ 70)
n = 29
Borderline CP at T1
but CP later still
prior age 10
(T-score ≥ 70)
n = 19
CP at T1
(T-score ≥ 70)
n = 274
CP at T1
(T-score ≥ 70)
n = 54
Borderline CP at T1
but no CP later
(T-score < 70)
n = 22
No CP
(T-score < 70)
n = 67
Temcheff, C.E., Martin-Storey, A., Lemieux, A., Latimer, E., & Déry, M. (2022). Trajectories of Medical Service Use among Girls and Boys with and without Early-Onset Conduct Problems. Frontiers in Psychiatry.
